# Dietary fat and breast cancer risk in the Swedish women's lifestyle and health cohort

**DOI:** 10.1038/sj.bjc.6604033

**Published:** 2007-10-16

**Authors:** M Löf, S Sandin, P Lagiou, L Hilakivi-Clarke, D Trichopoulos, H-O Adami, E Weiderpass

**Affiliations:** 1 Department of Medical Epidemiology and Biostatistics, Karolinska Institute, PO 281, Stockholm SE-171 77, Sweden; 2 Department of Hygiene and Epidemiology, University of Athens Medical School, 75 M Asias Street, Goudi GR-115 27, Athens, Greece; 3 Department of Epidemiology, Harvard School of Public Health, 677 Huntington Avenue, Boston MA 02115, USA; 4 Department of Oncology, Georgetown University, Washington, DC 20057, USA; 5 The Cancer Registry of Norway, Montebello N-0310, Oslo, Norway; 6 Department of Genetic Epidemiology, Samfundet Folkhalsan, PB 211, Helsinki Fi00250, Finland

**Keywords:** breast cancer, total dietary fat, monounsaturated fat, polyunsaturated fat, saturated fat, cohort

## Abstract

We investigated whether dietary intakes of total fat, monounsaturated fat (MUFA), polyunsaturated fat (PUFA) and saturated fat (SFA) were associated with breast cancer risk in a prospective cohort of 49 261 Swedish women (30–49 years at enrolment), which yielded 974 breast cancer cases by December 2005. Further, we evaluated if associations differed by oestrogen and/or progesterone receptor tumour status. Total fat, MUFA, PUFA or SFA were not associated with risk overall. However, women in the highest MUFA and PUFA quintile intake had a reduced breast cancer risk after age 50 years (hazard ratios: 95% confidence interval=0.45: 0.25–0.99 and 0.54: 0.35–0.85, respectively) compared to women in the lowest quintile. The associations did not differ by oestrogen or progesterone receptor status. Despite the negative findings, type of fat during premenopausal years may have later differential effects on risk.

Animal, ecological and some case–control studies suggest that a high consumption of total dietary fat increases breast cancer risk (Rose, 1997; Boyd *et al*, 2003), but results from cohort studies, mainly in postmenopausal women, are inconsistent (Smith-Warner *et al*, 2001; Boyd *et al*, 2003). Two large prospective US cohorts of postmenopausal women reported no (Kim *et al*, 2006) and a positive (Thiebaut *et al*, 2007) association. Conflicting results have also been reported regarding fat subtypes. Olive oil, rich in monounsaturated fat (MUFA), has been reported to reduce breast cancer risk in Mediterranean populations (Trichopoulou, 1995). There is another experimental evidence suggesting that MUFA, especially oleic acid, is protective (Bartsch *et al*, 1999); however of the 13 prospective studies only two reported an inverse association with MUFA intake (Wolk *et al*, 1998; Voorrips *et al*, 2002), both mostly of postmenopausal women in North European populations (Sweden and the Netherlands).

Risk factors may differ by hormone receptor status (Colditz *et al*, 2004), and an inverse association with fruit and vegetables has been reported for oestrogen-receptor negative (ER−) but not for ER+ tumours (Fung *et al*, 2005). Higher fat intake was slightly, but not significantly, positively associated with ER^+^/PR^+^ but negatively with ER^−^/PR^−^ tumours in postmenopausal women (Kushi *et al*, 1995). Among premenopausal women, stronger positive associations were reported between animal fat intake and risk of ER+ or PR+ tumours than with ER− or PR− tumours (Cho *et al*, 2003). In the randomised Women's Health Initiative study, the low-fat group showed a significantly lower risk of ER+ and PR− breast cancers (Prentice *et al*, 2006). In contrast, the associations with fat did not differ by ER or PR status in the Nurses' Health Study (Kim *et al*, 2006).

In a prospective cohort study in Swedish women, we investigated whether intakes of total dietary fat, MUFA, polyunsaturated fat (PUFA) and saturated fat (SFA) were associated with breast cancer risk.

## MATERIALS AND METHODS

The study cohort consisted of 49 261 women (Kumle *et al*, 2002). Briefly, some 96 000 women aged 30–49 years (born 1942–1962) and resident in the Uppsala Health Care Region in 1991–1992 were randomly selected from four age strata (30–34, 35–39, 40–44 and 45–49 years) and invited to participate in the Swedish component of the Scandinavian Women's Lifestyle and Health Cohort.

Around 49 261 women returned the study questionnaires and were enrolled in the study, of whom 9% were postmenopausal. We excluded 4692 women from the initial cohort due to breast cancer before enrolment (*n*=244), emigration before the follow-up (*n*=7), very high- or low-energy intakes <1 or 99% (*n*=1072) and missing information on BMI, education, use of oral contraceptives (OC), age at menarche, parity, age at first birth or breast cancer in first-degree relative (mother or sister) (*n*=3369). Thus, the final analysis was conducted on 44 569 women. The study was approved by the ethical committee at the University of Uppsala.

The self-administered questionnaire covered breast cancer risk factors, including average intake of foods and beverages (Lagiou *et al*, 2006). Dietary habits during the 6 months preceding enrolment were ascertained through a validated food-frequency questionnaire that also covered intake quantity of about 80 food items and beverages (Wolk *et al*, 1998). Individual intake of energy, total fat, MUFA, PUFA, SFA and alcohol were calculated by linking the amount of foods reported to the National Food Administration database (1989).

Follow-up was achieved through linking the study database with nationwide health registers in Sweden using the individually unique national registration number. From the total population registers, we received information on the dates of death and emigration. The national cancer registry, which began in 1958, identified prevalent cancers at enrolment and on incident cancers diagnosed during follow-up.

The start of follow-up was defined as the date of return of the questionnaire. Observation time was calculated from date of entry into the cohort until the date of breast cancer, emigration, death or 31 December 2005, whichever came first. Oestrogen receptor (ER− or ER+) and PR status (PR− or PR+), determined by means of an Abbott immunoassay (Pousette *et al*, 1986), was obtained by linkage with the regional cancer registry in Uppsala and was available for cases reported until 31 December 2004.

### Statistical analysis

The risk of breast cancer was analysed by fitting of Cox proportional hazard regression and the corresponding Poisson models using attained age as time scale (Korn *et al*, 1997). Hazard ratios (HRs) were considered significant when the associated two-sided 95% Wald-type confidence interval (CI) did not include unity, corresponding to a two-sided 5% level of significance. The goodness of fit of the different models was compared using the Akike Information Criteria (AIC).

For total fat, we fitted a model including education categories: 0–10, 11–13, >13 years + BMI categories: <25, 25–29.9, ⩾30 kg/m^2^ + Parity: 0, 1, 2, 3, ⩾4 +Age at menarche, years + use of OC: never, former and current + Age at first birth, years (Trichopoulos *et al*, 1983) + breast cancer in first-degree relative: yes or no + alcohol intake, in g/day + non-alcohol energy intake, in kJ/day. Total fat (10 g/day) was then added to the model, alternatively, as: (1) a continuous covariate, (2) in quintiles of total fat and (3) through splines. The models utilising splines allow for an informal evaluation of the functional form, for instance if a linear response function if appropriate. We tested for linear trend across fat and fat subtype quintiles using the median within each quintile.

For fat subtypes, we fitted models utilising the linear total fat model and adding each fat subtype, alternatively, as a linear continuous variable, as quintiles or as splines. Total fat was included because it can be a confounder on its own and changes in one type of fat were expected to reflect changes in the other two types. We also fitted a model with total unsaturated fat (MUFA+PUFA) and SFA as continuous linear variables excluding total fat. We used the standard method for energy adjustment (Willett *et al*, 1997). Total fat and fat subtypes were also evaluated as residuals on non-alcohol energy intake (residual method; Willett *et al*, 1997). Because these results were, as expected, similar compared to those obtained when using the standard model, we only report results from the latter.

The models above examine the effect of substitution of fat (or fat subtypes) for carbohydrate or protein (or fat subtype) when non-alcohol energy intake is held constant. In an additional model, we also adjusted for protein and carbohydrates by fitting the following variables: non-nutritional covariates, alcohol, protein, carbohydrate, PUFA, MUFA and total fat. It examines the effect of replacing SFA with PUFA or MUFA while keeping carbohydrate, protein and total fat (and thus energy) constant. We checked the proportional hazards assumption by plotting the Schoenfeld residuals *vs* time (Grambsch and Therneau, 1994).

We also tested if the associations with total fat, MUFA, PUFA and SFA differed by ER and PR status. We did not conduct any analyses with joint ER and PR status because there was a strong correlation between ER and PR status.

We do not have information about menopausal status after the start of follow-up. Based on the average age at menopause in Sweden being 50 years (Weiderpass *et al*, 1999), the effect of menopausal status and other risk factors in different periods of life were evaluated by fitting separate models for breast cancer occurring before and after the age of 50 years. All statistical analyses were carried out using the SAS software version 9.1.3 (SAS Institute Inc., Cary, NC, USA) and R version 2.5.0 (www.r-project.org).

## RESULTS

The 44 569 women were followed for an average of 13 years yielding a total of 615 988 person-years, during which 974 invasive breast cancers were reported to the cancer registry. The baseline characteristics for the entire cohort and by quintile of total dietary fat intake are shown in Table 1. The main food sources of MUFA were meat (30%), fat for food preparation and sandwiches (20%) and dairy products (20%). The corresponding figures for SFA were dairy products (40%), meat (20%) and fat for food preparation and sandwiches (15%), and for PUFA fat for food preparation and sandwiches (30%), meat (15%), cereal products (20%) and fish (7%).

Established non-nutritional risk factors were generally evident and, for example, parous had a lower risk than nulliparous women (e.g. parity=2 *vs* parity=0; HR: 0.77, 95% CI: 0.64–0.93), while having an affected first-degree relative showed an increased risk (HR: 1.67, 95% CI: 1.33–2.10).

Of the 974 breast cancer cases, 432 occurred before the age of 50 years. Analysing the time course starting at age 50 years, 14 437 women were censored (432 had breast cancer before the age 50 years, 295 died, 527 emigrated and 13 183 were too young to reach the age 50 years at the end of follow up) leaving 30 132 women with 542 breast cancer events for the analyses after age 50 years.

Table 2 shows breast cancer risks as HRs with 95% CIs for total fat, MUFA, PUFA and SFA for the entire cohort as well as before or after 50 years of age. Total fat intake as a linear continuous covariate (for a 10 g/day increase) or fitted as quintiles was not associated with overall risk or before or after age 50 years or when we fitted total fat as splines (data not shown). MUFA, PUFA or SFA was not associated with risk for the entire cohort nor before 50 years of age, irrespective of the models fitted.

Analysed as a continuous variable, the HR for MUFA intake was 0.55 (95% CI: 0.28–1.09) for a 10 g/day increase, while for SFA it was 1.45 (95% CI: 0.99–2.12), and for PUFA 0.58 (95% CI: 0.32–1.05) for breast cancer after age 50 years (Table 2). A linear form for the associations with MUFA, PUFA and SFA was supported by models using splines.

Compared to women in the first quintile of MUFA intake, women in the fifth quintile had a statistically significant decreased breast cancer risk (HR: 0.45; 95% CI: 0.25–0.99) for breast cancer after 50 years of age. There was a statistically significant trend for a decreased risk across the quintiles of MUFA intake (*P*=0.01). When the highest PUFA quintile was compared to the lowest, the HR was 0.54 (95% CI 0.35–0.85). When we finally combined MUFA and PUFA into unsaturated fat, the HR for breast cancer was 0.79 (95% CI: 0.62–1.01) for unsaturated fat for a 10 g/day increase and 1.21 (95% CI: 1.00–1.45) for saturated fat for a 10 g/day increase. When we adjusted for protein and carbohydrate in our additional model, lower but nonsignificant breast cancer risks were found when replacing SFA with PUFA (HR: 0.64, 95% CI: 0.31–1.30) or MUFA (HR: 0.62, 95% CI: 0.33–1.16) for a 10 g/day increase.

The point estimates in Table 2 suggest that there is a differential effect of MUFA, PUFA and SFA intakes on breast cancer risk occurring before or after 50 years of age. Thus, we also conducted tests for the interaction between age at diagnosis (<50 *vs* ⩾50 years) and intake of MUFA, PUFA, SFA as well as unsaturated fat (PUFA+MUFA). A statistical significant interaction with age at diagnosis (<50 *vs* ⩾50 years) was found for intakes of PUFA (*P*=0.049), SFA (*P*=0.048) and MUFA+PUFA (*P*=0.040), but not for MUFA (*P*=0.116). These results suggest that the effects of consumption of different types of dietary fat are mainly observed in breast cancer diagnosed after age 50 years.

### Hormone receptor status

For the 974 breast cancer cases, 559 (57%) were ER+, 160 (17%) were ER− and 255 (26%) had unknown ER tumour status. With respect to progesterone receptors, 474 (49%) were PR+, 235 (24%) PR− and 265 (27%) had unknown PR tumour status. A total of 94 cases had unknown ER and PR as they were registered during 2005 when no ER/PR status details were available to us. The distribution of ER+, ER−, PR+ and PR− was similar across MUFA, PUFA, SFA and total fat quintiles.

Overall, compared to women in the first quintile of PUFA intake, women in the fifth quintile were at a decreased risk of developing ER+ tumours (HR: 0.60, 95% CI: 0.39−0.92) and PR+ tumours (HR: 0.59, 95% CI: 0.37−0.95) when considering the entire cohort. Similar associations were found in the fifth quintile of PUFA intake compared to the first quintile for risk after age 50 years (HR: 0.55, 95% CI: 0.32–0.95) for ER+ tumours and: 0.52, 95% CI: 0.28–0.98) for PR+ tumours. However, there was no significant interaction between PUFA intake and age at diagnosis (<50 *vs* ⩾50) for ER+ tumours (*P*=0.72) or PR+ tumours (*P*=0.85). No other statistically significant associations between total fat, MUFA, PUFA and SFA intake and risk for ER+, PR+, ER− or PR− tumours were found in the entire cohort or before or after age 50 years, irrespective of the models used (data not shown).

## DISCUSSION

Our study did not find evidence for the entire cohort of an association between total fat, MUFA, PUFA or SFA intakes and breast cancer risk, or with ER or PR status. However, possible differential effects of type of fats during premenopausal years were suggested on risk above the age of 50 years. The lack of associations with total fat, MUFA, PUFA or SFA is in agreement with many earlier cohort studies (e.g. Kim *et al*, 2006).

However, statistically significant positive associations were recently found for total fat both in a meta-analysis (Boyd *et al*, 2003) and a cohort study (Thiebaut *et al*, 2007); also for MUFA, PUFA (Thiebaut *et al*, 2007) and for SFA in both these studies. All this may reflect differences between the populations studied. For example, Thiebaut *et al* studied women aged about 62 years at entry and followed them for 5.2 years on average. Our women were premenopausal at entry when their fat intake was assessed, and were followed for 13 years on average. Further, Thiebaut's women were mostly overweight, while the average BMI in our study was below 25 kg m^−2^. Finally, the intake ranges of total fat and subtypes were narrower in our study and the food sources for the subtypes slightly different.

The protective effect of MUFA in older women support is consistent with the Swedish mammography cohort on postmenopausal women (Wolk *et al*, 1998) but not with a nested case–control (Wirfalt *et al*, 2002) and several cohort studies, which, with some exceptions (Holmes *et al*, 1999; Voorrips *et al*, 2002), have reported no or even a positive association with MUFA (Velie *et al*, 2000; Smith-Warner *et al*, 2001; Boyd *et al*, 2003; Cho *et al*, 2003; Kim *et al*, 2006; Thiebaut *et al*, 2007). We also found a protective effect of PUFA in older women, as in a few case–control studies (Boyd *et al*, 2003) but not with any earlier prospective cohorts (Boyd *et al*, 2003; Kim *et al*, 2006; Thiebaut *et al*, 2007). Our results suggest that MUFA, PUFA and SFA might have different effects on risk between breast cancer occurring before and after age 50 years, for which no explanation is apparent; further work is indicated.

The most important limitation of our study was that dietary intake was assessed only once, involving misclassification among those who changed their dietary pattern during follow-up. Because this is likely to be nondifferential, it would attenuate the strength of any true association. However, there is a potential risk that the degree of this misclassification increases over time because women change their dietary habits. Thus, the longer the follow-up, the more attenuated the association might be and we found some evidence for this: thus among women over age 50 years, the risk associated with MUFA (as a linear continuous variable) was 0.35 (95% CI: 0.13–0.97) for follow-up 0–10 years, but 1.00 (95% CI: 0.40–2.54) for longer follow-up; there was no significant interaction between MUFA and follow-up time (*P*=0.44).

Women with a high PUFA intake had a decreased risk of developing ER+ and PR+ tumours after age 50 years, while no other association with fat or its subtypes differed by ER or PR status. An increased risk for ER+/PR+ tumours with higher total fat (Kushi *et al*, 1995) or those with animal fat intake (Cho *et al*, 2003) or those with no association has been reported (Kim *et al*, 2006). We cannot rule out that our findings are due to chance since the numbers in each category (ER+, PR+, PR− and ER−) was low and we made many comparisons. However, they might be explained by PUFA containing relatively high levels of *n*-3 PUFA if these, as reported, reduce the incidence of ER− breast tumours (Hislop *et al*, 1988).

High fat intake may increase circulating oestrogens levels (Wu *et al*, 1999) which then promote the growth of malignant mammary cells. Saturated fat intake may increase risk through multiple mechanisms, for example through their high levels of hormone-like substances that were fed to cattle (Mitra *et al*, 2004), by increasing the expression of genes promoting inflammatory responses and inhibiting apoptosis, such as NF-kB (Lee *et al*, 2001) or by increasing levels of cholesterol and low-density lipoprotein which promote growth of malignant mammary cells (Le Guevel and Pakdel, 2001). Since PUFA and MUFA intakes have been reported to increase, reduce or have no effect on risk, invoking a biological mechanism is premature.

Strengths of our study include its prospective design, large size and complete follow-up. Cancer registration in Sweden is obligatory, making the assessments of cases virtually complete. Furthermore, we were able to adjust for several risk factors for breast cancer. Misclassification of fat intakes due to measurement error in the food frequency questionnaire is unavoidable, but given the study design likely to be nondifferential, and thus attenuating any true association. A recent study found a null association with total fat intake using food frequency questionnaire data but a positive association when a 7-day food diary was used (Bingham *et al*, 2003). A positive finding may therefore depend on the dietary method used.

Our study provides no evidence that total fat, MUFA, PUFA or SFA is associated with overall breast cancer risk, but does not rule out the possibility that types of fat may have differential effects on breast cancer risk before and after age 50 years.

## Figures and Tables

**Table 1:** Baseline characteristics for the entire cohort at enrolment as well as by quintile of total dietary fat intake

		**Total fat quintiles**				
	**Entire cohort**	**1**	**2**	**3**	**4**	**5**
*Characteristics*						
*N*	44 569	8699	8950	8947	9025	8948
Age, mean±s.d. (years)	39±3	41±6	40±6	39±6	39±6	38±6
*BMI, n* (%)(*kg m*^−*2*^)						
<25	32 329 (72)	6003 (69)	6347 (71)	6518 (73)	6707 (74)	6754 (75)
25–30	9668 (22)	2129 (24)	2074 (23)	1958 (22)	1817 (20)	1690 (19)
⩾30	2572 (6)	567 (7)	529 (6)	471 (5)	501 (6)	504 (6)
						
*Education, n* (%) *(years)*						
up to 10	13 149 (30)	3130 (36)	2824 (32)	2556 (29)	2395 (26)	2244 (25)
11–13	17 478 (39)	3144 (36)	3443 (38)	3583 (40)	3660 (41)	3648 (41)
>13	13 942 (31)	2425 (28)	2683 (30)	2808 (31)	2970 (33)	3056 (34)
						
*Use of oral contraceptives, n* (%)						
Never	7213 (16)	1467 (17)	1415 (16)	1372 (15)	1423 (16)	1536 (17)
Former	31 577 (71)	6220 (71)	6379 (71)	6364 (71)	6433 (71)	6181 (69)
Current	5779 (13)	1012 (12)	1156 (13)	1211 (14)	1169 (13)	1231 (14)
						
*Parity, n* (%)						
0	6146 (14)	1470 (17)	1279 (14)	1183(13)	1105 (12)	1109 (12)
1	6870(15)	1429 (16)	1421 (16)	1356 (15)	1340 (15)	1324 (15)
2	19 328 (43)	3638 (42)	3950 (44)	4001 (45)	3951 (44)	3788 (42)
3	9264 (21)	1615 (19)	1779 (20)	1824 (20)	2009 (22)	2037 (23)
⩾4	2961 (7)	547 (6)	521 (6)	583 (7)	620 (7)	690 (8)
						
*Alcohol intake, n* (%) (*g*/*day)*						
<5	33 338 (74.8)	6591 (75.8)	6746 (75.4)	6605 (73.8)	6705 (74.3)	6691 (74.8)
5–25	11 039 (24.8)	2068 (23.8)	2162 (24.1)	2308 (25.8)	2283 (25.3)	2218 (24.8)
>25	192 (0.4)	40 (0.4)	42 (0.5)	34(0.4)	37 (0.4)	39 (0.4)
						
*Breast cancer in first-degree relative, n* (%)						
Yes	2036 (5)	413 (5)	394 (4)	420 (5)	430 (5)	379 (4)
No	42 533 (95)	8286 (95)	8556 (96)	8527 (95)	8595 (95)	8569 (96)
						
Energy, mean±s.d. (kJ/day)	6290±1820	4330±1080	5530±890	6400±890	7320±960	8920±1270
						
*Carbohydrates, mean*±*s.d.*						
g/day	189±59	142±48	172±44	195±43	217±47	252±51
% energy	51±16	56±19	53±14	52±11	50±11	48±10
						
*Protein, mean*±*s.d.*						
g/day	61±19	43±12	54±11	62±12	70±13	84±16
% energy	16±5	17±5	17±3	16±3	16±3	16±3
						
*Total fat, mean*±*s.d. (g*/*day)*						
g/day	52±19	29±6	43±3	53±3	63±4	84±12
% energy	31±11	25±5	29±2	31±2	32±2	35±5

s.d.=standard deviation.

%energy=percent energy of total energy intake.

**Table 2:** Risk of breast cancer estimated as hazard ratios (HR) with 95% confidence intervals (CIs) for total fat, MUFA, PUFA and SFA for the entire cohort as well as for breast cancers occurring before or after 50 years of age

	**Quintiles**							
	**Q1**	**Q2**	**Q3**	**Q4**	**Q5**	**Trend over categories (*P*)**	**Intake of continuous form per 10 g/day**	***P***
Entire cohort^a^								
*Total fat*								
Median, g/day	30.8	42.9	52.4	63.1	80.7			
(% energy)	(26)	(29)	(31)	(32)	(34)			
Cases	208	198	193	206	169			
HR (95% CI)^b^	1.00	0.99 (0.80–1.22)	1.01 (0.80–1.28)	1.13 (0.86–1.47)	1.02 (0.72–1.45)	0.70	1.04 (0.97–1.11)	0.32
								
*MUFA*								
Median, g/day	10.4	14.4	17.5	21.0	26.5			
(% energy)	(9)	(10)	(10)	(11)	(11)			
Cases	206	202	205	189	172			
HR (95% CI)^b^	1.00	0.98 (0.79–1.23)	1.01 (0.76–1.33)	0.95 (0.67–1.35)	0.88 (0.53–1.46)	0.65	0.82 (0.49–1.35)	0.43
								
*PUFA*								
Median, g/day	4.3	6.0	7.3	8.7	11.2			
(% energy)	(3.6)	(4.0)	(4.2)	(4.4)	(4.8)			
Cases	209	201	191	205	168			
HR (95% CI)^b^	1.00	0.93 (0.76–1.14)	0.89 (0.71–1.12)	0.92 (0.71–1.19)	0.72 (0.52–1.00)	0.08	0.83 (0.54–1.27)	0.40
								
*SFA*								
Median, g/day	12.9	18.6	23.3	28.7	37.9			
(% energy)	(11)	(12)	(14)	(15)	(16)			
Cases	207	198	198	188	183			
HR (95% CI)^b^	1.00	0.98 (0.78–1.23)	1.04 (0.79–1.36)	1.02 (0.73–1.44)	1.12 (0.69–1.81)	0.65	1.12 (0.84–1.49)	0.43
								
Breast cancer before the age 50 years^c^								
*Total fat*								
Median, g/day	30.8	42.9	52.4	63.1	80.7			
(% energy)	(26)	(29)	(31)	(32)	(34)			
Cases	78	83	82	99	90			
HR (95% CI)^b^	1.00	1.10 (0.79–1.52)	1.12 (0.78–1.62)	1.44 (0.95–2.16)	1.46 (0.87–2.47)	0.10	1.06 (0.96–1.18)	0.22
								
*MUFA*								
Median, g/day	10.4	14.4	17.5	21.0	26.5			
(% energy)	(9)	(10)	(10)	(11)	(11)			
Cases	76	84	93	84	95			
HR (95% CI)^b^	1.00	1.16 (0.81–1.66)	1.35 (0.88–2.06)	1.30 (0.76–2.21)	1.69 (0.81–3.51)	0.20	1.31 (0.63–2.73)	0.47
								
*PUFA*								
Median, g/day	4.3	6.0	7.3	8.7	11.2			
(% energy)	(3.6)	(4.0)	(4.2)	(4.4)	(4.8)			
Cases	78	80	94	97	83			
HR (95% CI)^b^	1.00	0.99 (0.71–1.38)	1.16 (0.82–1.66)	1.20 (0.80–1.78)	1.06 (0.64–1.75)	0.71	1.31 (0.79–2.46)	0.40
								
*SFA*								
Median, g/day	12.9	18.6	23.3	28.7	37.9			
(% energy)	(11)	(12)	(14)	(15)	(17)			
Cases	84	83	85	86	94			
HR (95% CI)^b^	1.00	0.93 (0.66–1.30)	0.90 (0.60–1.35)	0.88 (0.54–1.45)	0.93 (0.56–1.88)	0.86	0.81 (0.53–1.23)	0.32
								
Breast cancer after the age 50 years^d^								
*Total fat*								
Median, g/day	30.8	42.8	52.4	63.0	80.2			
(% energy)	(27)	(29)	(31)	(32)	(34)			
Cases	130	115	111	107	79			
HR (95% CI)^b^	1.00	0.91 (0.70–1.20)	0.93 (0.69–1.27)	0.93 (0.65–1.33)	0.76 (0.47–1.22)	0.34	1.01 (0.93–1.11)	0.77
								
*MUFA*								
Median, g/day	10.4	14.4	17.5	20.9	26.4			
(% energy)	(9)	(10)	(10)	(11)	(11)			
Cases	130	118	112	105	77			
HR (95% CI)^b^	1.00	0.86 (0.64–1.15)	0.79 (0.55–1.15)	0.73 (0.46–1.17)	0.45 (0.25–0.99)	0.01	0.55 (0.28–1.09)	0.09
								
*PUFA*								
Median, g/day	4.3	6.0	7.3	8.7	11.2			
(% energy)	(3.6)	(4.0)	(4.3)	(4.5)	(4.9)			
Cases	131	121	97	108	85			
HR (95% CI)^b^	1.00	0.89 (0.68–1.15)	0.72 (0.53–0.98)	0.76 (0.54–1.06)	0.54 (0.35–0.85)	0.08	0.58 (0.32–1.05)	0.06
								
*SFA*								
Median, g/day	12.9	18.6	23.3	28.8	37.6			
(% energy)	(11)	(13)	(14)	(15)	(16)			
Cases	123	115	113	102	89			
HR (95% CI)^b^	1.00	1.03 (0.77–1.39)	1.16 (0.80–1.67)	1.15 (0.72–1.83)	1.29 (0.66–2.50)	0.44	1.45 (0.99–2.12)	0.06

% energy=percent energy of total energy intake.

a *n*=44 569; 974 breast cancer cases.

b Adjusted for education, parity, age at menarche, use of oral contraceptives, age at first birth by parity, first-degree relative with breast cancer, non-alcohol total energy intake, total fat intake, BMI and alcohol intake. Attained age was utilised as time scale.

c *n*=44 569; 432 breast cancer cases.

d *n*=30 132; 542 breast cancer cases.
